# Readability of Online Patient Educational Materials for Rosacea: Systematic Web Search and Analysis

**DOI:** 10.2196/67916

**Published:** 2025-09-08

**Authors:** Derek Nguyen, Jennifer Javaheri, Daniel Nguyen, Vy Han

**Affiliations:** 1 University of California Riverside, 900 University Avenue, Riverside, CA, 92521, United States, 1 7148802250; 2 California University of Science and Medicine, Colton, CA, United States

**Keywords:** rosacea, websites, Google, readability, patient information, health education, dermatology, health literacy

## Abstract

Most online educational materials about rosacea exceed recommended readability levels, often requiring at least a high school education to understand, with content authored by physicians being significantly more difficult to read than that written by nonphysicians.

## Introduction

Rosacea is a common skin condition characterized by persistent facial flushing and redness, which can progress to visible blood vessels and pus-filled bumps. Patients with rosacea often struggle to manage their symptoms and the psychosocial burdens associated with the condition [1]. As a result, many seek information beyond their health care providers to understand the disease better [1]. With increased internet accessibility, dermatological patients often consult online health resources when making decisions on treating illnesses [2]. Since the average reading ability of a US adult is at the eighth-grade level, the American Medical Association recommends patient educational material be written at a sixth-grade reading level [2]. Adhering to these readability standards is essential for improving patient comprehension and ensuring that dermatological resources are accessible to a wider audience. In this study, we assess the readability of available online educational material dedicated to the management and treatment of rosacea.

## Methods

A web search was conducted on April 25, 2025, using Google with the query “Rosacea Patient Information.” The top 50 results were selected for the study as past research suggests that internet users generally do not search beyond this point [3]. The contents of these websites were then evaluated by 2 reviewers for relevance to patient education, with non-English sites and advertisements excluded. In total, 43 websites passed the inclusion criteria. The readability of their contents was assessed using an online scoring tool [4]. Seven established reading tools (Flesch Reading Ease, Flesch-Kincaid Grade Level, Gunning Fog Index, Coleman-Liau Readability Index, SMOG [Simple Measure of Gobbledygook] index, Automated Readability Index [ARI], and Linsear Write Readability Formula) were used in this process [5]. These tests assess various factors, including word count, character count, syllable count, and complexity, to generate a composite score that corresponds to a specific reading level. Finally, we compared group differences in readability between doctor of medicine (MD) and non-MD authors. Bachelor of medicine, bachelor of surgery, and other equivalent medical degrees were included in the MD category.

Readability scores were analyzed using 2-tailed *t* tests, with a *P* value <.05 considered statistically significant.

## Results

Despite guidelines recommending a sixth-grade reading level for patient materials, few websites on rosacea met this standard across commonly used readability formulas: 3 for ARI, 5 for Flesch-Kincaid Grade Level, 4 for SMOG, 0 for the Coleman-Liau Index, 0 for the Gunning Fog Index, 4 for the Linsear Write Readability Formula, and 0 for Flesch Reading Ease. Furthermore, online educational material on rosacea exceeded this guideline by an average of 4.2 grade levels (Table 1). The differences between articles written by MD authors and non-MD authors were further analyzed, with articles on rosacea written by MD authors (n=18) found to require higher literacy skills than articles written by non-MD authors (n=25) across all readability formulas (Table 2). Previous studies have found a similar effect, highlighting a trend in which materials authored by health care professionals tend to be more complex and less accessible to the general public [6,7]. Interestingly, we observed a diverse range of authorship across the articles, with contributions from various organizations, including medical societies, academic centers, and online platforms, featuring both MD and non-MD authors.

**Table 1.** Readability of rosacea online materials according to website category.

Readability	All websites (n=43), mean score (SD; range)	Online resources (n=21), mean score (SD; range)	Academic (n=12), mean score (SD; range)	Medical societies (n=10), mean score (SD; range)
Automated Readability Index	9.7 (2.9; 4.8‐17.1)	9.9 (3.2; 4.8‐17.1)	8.9 (3.0; 5.3‐16.3)	10.4 (2.0; 7.3‐14.2)
Gunning Fog Index	11.1 (2.5; 6.1‐16.3)	11.1 (2.9; 6.1‐16.3)	10.4 (2.3; 6.5‐14.8)	11.9 (1.7; 9.6‐14.9)
Coleman-Liau Readability Index	11.6 (2.6; 7.2‐17.8)	11.6 (2.8; 7.2‐17.6)	11.3 (2.8; 7.6‐17.8)	12.1 (2.1; 10.0‐17.1)
Simple Measure of Gobbledygook index	8.9 (2.0; 5.2‐13.9)	9.0 (2.3; 5.2‐13.9)	8.2 (1.9; 5.4‐12.2)	9.5 (1.1; 7.7‐11.2)
Linsear Write Readability Formula	9.9 (3.5; 4.1‐16.8)	10.1 (3.6; 4.1‐16.8)	9.6 (4.4; 4.1‐15.6)	9.6 (1.9; 7.4‐12.1)
Flesch-Kincaid Grade Level	9.2 (2.7; 5.0‐15.4)	9.4 (3.1; 5.0‐15.4)	8.4 (2.6; 5.2‐14.0)	9.8 (1.8; 7.3‐13.3)
Flesch Reading Ease	52 (15.6; 14‐75)	52 (17.5; 14‐73)	55.2 (14.6; 25‐75)	48.9 (13.1; 19‐62)

**Table 2.** Differences in readability of rosacea content: doctor of medicine (MD) versus non-MD authors.

Readability	MD (n=18), mean score (SD; range)	Non-MD (n=25), mean score (SD; range)	*P* value
Automated Readability Index	11.3 (3.0; 5.3‐17.1)	8.4 (2.1; 4.8‐13.7)	.002
Gunning Fog Index	12.8 (2.3; 8.2‐16.3)	9.7 (1.9; 5.0‐12.8)	<.001
Coleman-Liau Readability Index	13.1 (2.8; 7.6‐17.8)	10.5 (1.9; 7.2‐13.4)	.002
Simple Measure of Gobbledygook index	10.1 (1.8; 6.3‐13.9)	7.9 (1.6; 5.2‐11.7)	.007
Linsear Write Readability Formula	11.0 (3.3; 4.3‐16.8)	9.0 (3.5; 4.1‐14.6)	.03
Flesch-Kincaid Grade Level	10.8 (2.7; 5.3‐15.4)	7.9 (2.0; 5.0‐12.8)	.005
Flesch Reading Ease	42.6 (16.1; 14‐73)	59.4 (10.6; 41‐75)	.008

## Discussion

The results obtained from 7 well-known and validated readability scales suggest that readers need to have a minimum of a high school level understanding of the English language to understand the majority of available online material on rosacea. Patients encounter an additional barrier when seeking credible health information from physician authors, as many of these authors lack the knowledge to address health literacy concerns and are therefore unable to explain these topics to readers effectively [8]. Previous studies have shown that patients with lower health literacy are more likely to distrust information from physicians [9]. Consequently, as the internet becomes a primary source for health information, this gap in readability not only hinders health decision-making but also increases the risk of incorrect self-diagnosis and self-treatment, which can worsen health outcomes and contribute to unnecessary strain on health care resources [10]. The primary limitation of our study was that we did not assess the content quality or understandability of the websites. As a result, the findings are limited to the readability aspect alone and do not provide a comprehensive evaluation of the usefulness or accuracy of the information presented. Further studies can evaluate patient comprehension of online dermatology patient educational material. Effective communication of medical knowledge to the general public is essential to bridging the readability gap. Readability should be facilitated through interactions between physicians and readers online and better physician understanding of patients’ health literacy needs.

## Abbreviations

ARI: Automated Readability Index
MD: doctor of medicine
SMOG: Simple Measure of Gobbledygook
